# Comparative Study of Sun Compass Orientation in Migrating Anadromous versus Resident Freshwater Threespine Sticklebacks (*Gasterosteus aculeatus*)

**DOI:** 10.1093/iob/obaf022

**Published:** 2025-05-28

**Authors:** L Spiecker, M Laurien, F Schröder, J C C Moreno, S M Fübbeker, S Lüdtke, C Walter, A W Nolte, G Gerlach

**Affiliations:** Institute of Biology and Environmental Science, Carl von Ossietzky University Oldenburg, 26111 Oldenburg, Germany; Department of Biogeography, University of Trier, 54296 Trier, Germany; Institute of Biology and Environmental Science, Carl von Ossietzky University Oldenburg, 26111 Oldenburg, Germany; Institute of Biology and Environmental Science, Carl von Ossietzky University Oldenburg, 26111 Oldenburg, Germany; Institute of Biology and Environmental Science, Carl von Ossietzky University Oldenburg, 26111 Oldenburg, Germany; Institute of Biology and Environmental Science, Carl von Ossietzky University Oldenburg, 26111 Oldenburg, Germany; Institute of Biology and Environmental Science, Carl von Ossietzky University Oldenburg, 26111 Oldenburg, Germany; Institute of Biology and Environmental Science, Carl von Ossietzky University Oldenburg, 26111 Oldenburg, Germany; Institute of Biology and Environmental Science, Carl von Ossietzky University Oldenburg, 26111 Oldenburg, Germany; Institute of Biology and Environmental Science, Carl von Ossietzky University Oldenburg, 26111 Oldenburg, Germany

## Abstract

In the northern coastal hemisphere, different ecotypes of the threespine stickleback (*Gasterosteus aculeatus*) can be distinguished phenotypically by a different number of lateral bone plates and by their body shape and size. We focused on (1) anadromous sticklebacks, which migrate from the sea to rivers to spawn and (2) freshwater sticklebacks, which live in rivers all year round. Migration behavior is a key feature in the evolution of ecotypes, but the underlying mechanisms of migration are poorly understood. To learn more about possible orientation mechanisms that could lead to goal-directed migration, we tested anadromous sticklebacks for their sun compass orientation and compared their orientation behavior with that of the freshwater ecotype. Behavioral experiments revealed ecotype-dependent differences, whereby the ability to orient is consistently present in the anadromous ecotype, whereas the orientation in the freshwater ecotype corresponds to a random directional distribution.

## Introduction

Numerous fish species migrate from a few to thousands of kilometers to reach suitable spawning areas or feeding grounds. Migratory fish have developed elaborate abilities to detect a variety of sensory cues to integrate these signals within their nervous systems, and use them as part of highly efficient navigational strategies. Fish can be guided by acoustic (Radford et al. 2011), olfactory cues (Hasler and Wisby 1951; Atema et al. 2002; Gerlach et al. 2007), magnetic stimuli (see for review Formicki et al. 2019), and they can also use celestial cues such as a sun compass for orientation.

A time-compensated sun compass enables animals to orient themselves to the azimuthal position of the sun and compare this with the current time of day. With the help of their endogenous circadian rhythm, they can correctly interpret the position of the sun at any time of day and orient to a chosen direction. If the desired compass direction is south, they must move in the direction of the sun at noon, but at a 90° angle to the west of the sun at 6 pm. We showed a time-compensated sun compass in juvenile coral reef fish (*Ostorhinchus doederleini*) (Mouritsen et al. 2013), herring (*Clupea harengus*) (Spiecker et al. 2022), and sprats (*Sprattus sprattus*) (Laurien et al. 2024b). Since other visual orientation cues are rare due to the turbidity of the water and the often-muddy seabed, orientation by the sun seems to be a frequently used orientation mechanism. Here, we examined whether (1) threespine sticklebacks (*Gasterosteus aculeatus*) use a sun compass and (2) orientation of freshwater threespine sticklebacks differs from anadromous ones. Anadromous sticklebacks are found throughout the North Sea and are distributed in coastal areas (Wootton 1976) (see also DATRAS, the online database of the International Council for the Exploration of the Sea, ICES). Orientation requirements are particularly high for fish starting their migration in the open sea. The origin and migration routes of sticklebacks, which migrate to the rivers along the northwest German coast in spring to spawn, are unknown. Also their orientation capabilities are not understood.

The threespine stickleback is an exceptional model organism for divergent adaptations and parallel evolution (Rundle et al. 2000; McKinnon and Rundle 2002). Ancestral marine sticklebacks adapted to freshwater and colonized new environments multiple times since the end of the last ice age (Aguirre et al. 2022). Compared to anadromous sticklebacks, which typically have up to 36 lateral bony plates, freshwater sticklebacks show a lower number of plates on their body side (Schluter et al. 2004; Walker 2008). They are also smaller and differ in their body shape (Walker 2008). Such morphological changes are explained by a reduced predation risk, which is why most ecotypes can be identified visually. Evolution of reduced armor in freshwater populations is due to positive selection from both abiotic and biotic mechanisms (Barrett 2010). A major effect gene (ectodysplasin-A or *Eda*), along with several minor effect genetic regions, have been shown to control lateral plate variation (Colosimo et al. 2005).

We conducted orientation experiments to investigate whether anadromous sticklebacks and freshwater sticklebacks possess as dispersal traits a celestial sun compass and whether the ecotypes differ in orientation behavior.

## Materials and methods

### Statement on compliance with ethical standards

All animal procedures were approved by the Animal Care and Use Committees of the Niedersächsisches Landesamt für Verbraucherschutz und Lebensmittelsicherheit (LAVES, Oldenburg, Germany), Az.: 33.19-42502-04-17/2721 and were performed in accordance with the relevant guidelines and regulations.

### Sampling and holding

**Fig. 1.**
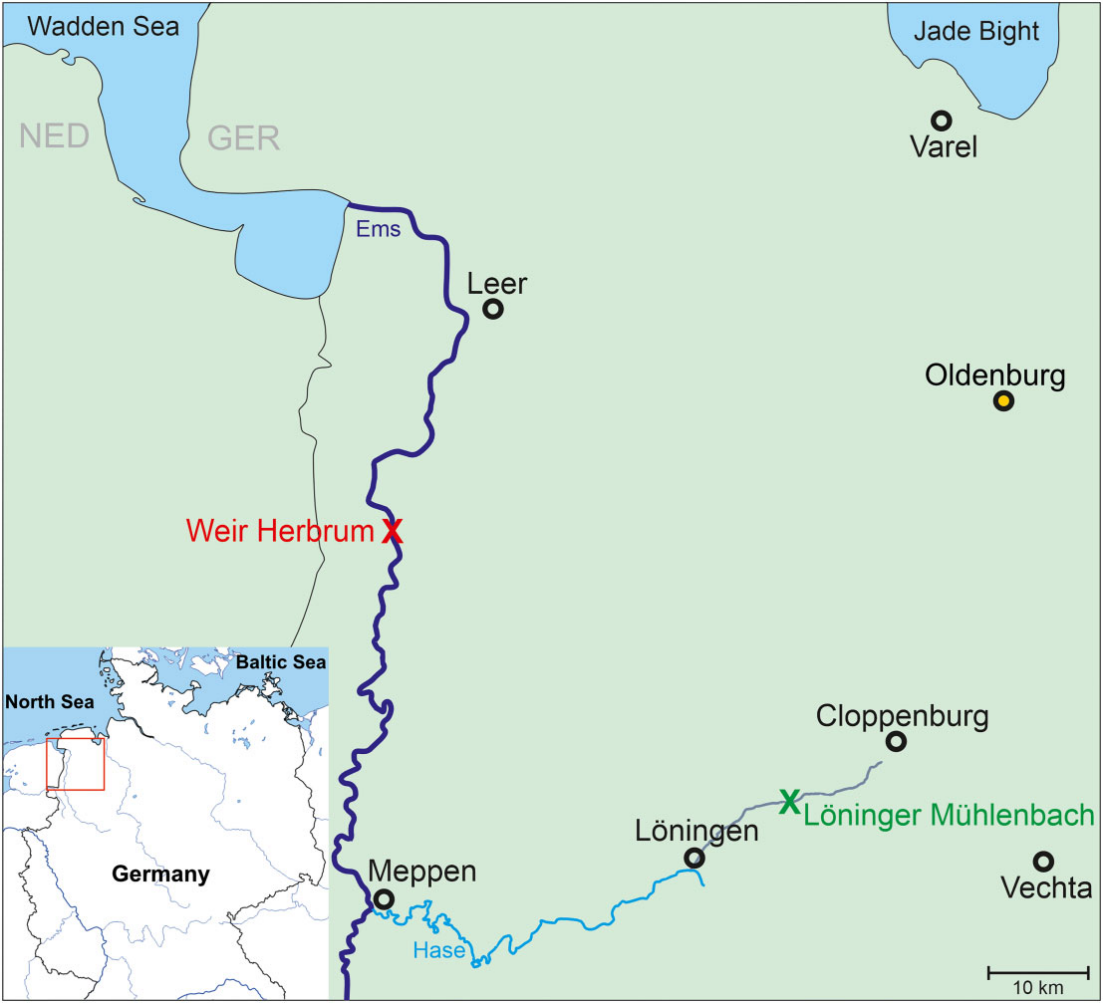
Map of the sampling area in Lower Saxony, northern Germany. Many rivers that flow into the Wadden Sea are known to be relevant areas for migrating sticklebacks—one of these rivers is the Ems (dark blue line). Anadromous sticklebacks can enter the Ems from the Wadden Sea, between the border of Germany (GER) and the Netherlands (NED) (gray line), and can follow the rivers path in southerly direction. Thin black line west of the River Ems indicates border between the Netherlands and Germany. The anadromous fish that were tested in Oldenburg were caught during their spawning migration in a fish ladder at Weir Herbrum (53.032008 N, 7.314717 E.The Wadden Sea and the Jade Bight are connected to the North Sea.

In April 2019 and 2021, freshwater sticklebacks were caught with hand nets at the Löninger Mühlenbach and at the River Ems (Fig. 1). Anadromous sticklebacks were collected during the peak of the spawning migrations at night at the river Ems at a fish ladder at Weir Herbrum (Fig. 1) in March and April 2020 and 2021. At high tide, the river Ems is temporarily influenced by saltwater from the North Sea. The exact salinity at the time of catching was not determined. The fish were transported to Oldenburg, where they were kept in 120- to 200-L glass aquariums filled with tap water from a reservoir tank. The water temperature of 15°C was regulated with a TECO Aquarium Cooler TK 500, and the water quality was ensured by filtration with an external filter (Eheim professional) and regular water tests to ensure constant conditions (pH 6.5–7.0, NO_3_ < 50 mG/L). Daily, after the experiments, the animals were fed with *Neomysis* sp.; they were kept under a light/dark cycle of 12:12 h. At the end of the yearly experiments, fish were euthanized using MS222 and preserved in ethanol for further phenotyping.

The Löninger Mühlenbach (gray line; catch site: (52.747444 N, 7.785917 E) is a <1 m broad freshwater stream, further inland compared to the Weir Herbrum. The rivers/streams Hase (turquoise line) form a connection with the Ems near the city Meppen; only freshwater inhabitants were found here identified by morphological differences (pers. obs. A. Nolte).

### Experimental design

All experiments were performed in Oldenburg, Germany, from March to May of 2019, 2020, and 2021. Anadromous fish caught during their migration period in 2020 and 2021 were analyzed for their orientation according to the sun compass (Sun); in 2021, a separate group of anadromous fish was time shifted (TS) by 6 h (TS) immediately after capture and the light was switched on at 01:30 am, light off 01:30 pm. TS fish were able to adapt to the new light regime for 7 days before behavioral orientation tests started. TS fish were tested between 07:30 am and 12:30 pm and Sun fish were tested between 08:30 am and 06:30 pm. All fish were tested between three and six times in the same condition. They were housed separately between tests. Multiple testing ensures an estimate of the orientation accuracy and consistency of the mean orientation direction. Freshwater fish were tested in the same way as described for Sun conditions, but they were not TS.

**Fig. 2.**
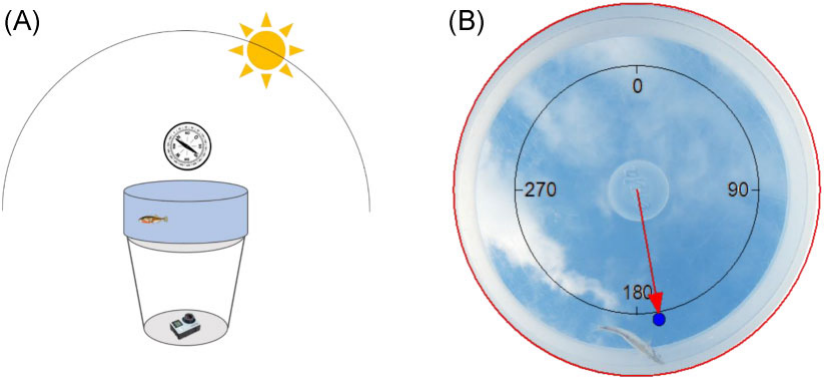
Experimental setup for sun compass testing. **(A)** An experimental bowl with 5 L of freshwater and one experimental animal was placed on top of a bucket in the field, ensuring a clear and unhindered view of the sky. A north-facing GoPro camera with a view of the sky was attached to the bottom of the bucket to record time-lapse videos of the test runs. **(B)** The position of the fish's head was determined every 30 s, for 40 observations per fish. 0° refers to North, 90° to East, 180° to South, and 270° to West.

For observing orientation behavior, sticklebacks were placed individually in testing bowls (diameter 30 cm, 7–8 cm water depth) containing 5 L of freshwater (temperature similar to tank water) and put on top of a bucket (see Fig. 2A). This bucket was placed near the university in an open field to ensure that the fish had a clear view of the sky with no visible landmarks. After a 5-min acclimatization period, every individual stickleback was tested for 20 min; a GoPro camera (GoPro Hero 4 silver) was placed on the bottom of the bucket recording a time-lapse video by taking a picture every 30 s (see example picture in Fig. 2B). The camera was aligned toward North.

### Ecotype determination

To distinguish between freshwater and anadromous ecotypes, the lateral bone plates of each fish, the presence of a bony keel (that is found close to the tail fin on the lateral side—connected to the most posterior bone plates) and the body size were determined. Freshwater sticklebacks are considerably smaller and have a reduced number of bony plates. The genetic basis for the phenotypic differences in numbers of bony plates has been attributed to the large-effect gene ectodysplasin (*Eda*) and its pleiotropic effects (Colosimo et al. 2004; Barrett et al. 2008; Archambeault et al. 2020). Anadromous sticklebacks are generally highly plated with up to 36 plates on each side and a characteristic keel anterior to the caudal fin (Myhre and Klepaker 2009).

After the experiments, we performed detailed analyses to classify all fish with >10 plates as anadromous saltwater ecotypes and 10 or less (“low plated”) as freshwater ecotypes (Hansen et al. 2016). In some cases, the plate count differed for each side of the body. If one side was fully plated and the other partially, we used the presence of a bony keel and the body size as an additional feature to determine the ecotype. Fish with a caudal keel were classified as anadromous ecotypes, while smaller ones, or individuals that lacked a caudal keel, were categorized as freshwater fish. All fish caught at the Löninger Mühlenbach in 2019 and some at the river Ems in 2021 belonged to the resident freshwater ecotype. Only those caught at the fish pass at the river Ems, which were unambiguously identified as anadromous fish (>10 bony plates and presence of a keel), were used for behavioral trials. The average sizes were 63.5 mm ± 0.79 SEM in 2020 and 61.6 mm ± 0.80 SEM in 2021 for anadromous fish from the Ems fish pass, while freshwater ecotypes from Löninger Mühlenbach in 2019 had an average body length of 35 mm ± 0.37 SEM. Since we do not have additional data on life history of these fish, the definition of the ecotype is based only on these described factors.

### Data analysis and statistics

Using ImageJ (Rasband, W.S., ImageJ, U.S. National Institutes of Health, Bethesda, MD, USA), the position of the fish's head was used as a proxy of orientation and the position was labeled in every 30 s for a total of 40 observations per fish (see Fig. 2). After conversion of the positional data into compass directions, directional orientation vectors were estimated using the circular statistics program Oriana4 (Kovach 2011) and plotted using the R package ggplot2 (Wickham et al. 2024) in R version 4.4.2. We used Rayleigh's Uniformity Test (Fisher 1993), which calculates the probability of the null hypothesis that the data are distributed in a uniform manner. To compare orientation of fish in Sun versus TS test, a Mardia–Watson–Wheeler test was used implemented in the program Oriana4.

First, we calculated the mean vector of orientation from the 40 frames captured from each experimental run for Sun and TS fish. Second, the mean direction of each individual fish was calculated by averaging all the mean vectors of the same fish during multiple trials. This method provides an average direction per individual. Third, we determined the group's mean orientation by combining all individuals of the same ecotype. We pooled data from 2 years (Sun, 2020 and 2021) after finding that they were statistically similar to each other (Mardia–Watson–Wheeler test implemented in Oriana). TS experiments were conducted in 2021 only because we did not catch enough fish in 2020 for both experiments. This method assumes a 15° change in sun azimuth per hour, which adds up to a 90° shift in 6 h. Thus, we expected a 90° difference in the orientation of Sun and TS fish.

The orientation consistency of the fish is indicated by the length of the mean vector “*r*,” (*r* is ranging from 0 to 1, *r *= 0 means no directedness and *r* = 1 the fish is always pointing to the same direction). To determine whether the degree of cloud cover had an effect on the orientation ability of the fish, we compared the length of the mean *r* vectors at different cloud covers during the test. *R*-vectors of all test series of Sun were calculated according to increasing cloud cover conditions (categories = 0–20%, >20–40%, >40–60%, >60–80%, and >80–100%). Three subjects independently determined the degree of cloud cover by rating the photos taken with the GoPro pointed at the sky during the experiments (see also Fig. 2). We used a general linear mixed model for an overall comparison implemented in R (version 4.4.2) to analyze the impact of cloud coverage on orientation directedness.

## Results

### Sun compass orientation ability and direction

**Fig. 3.**
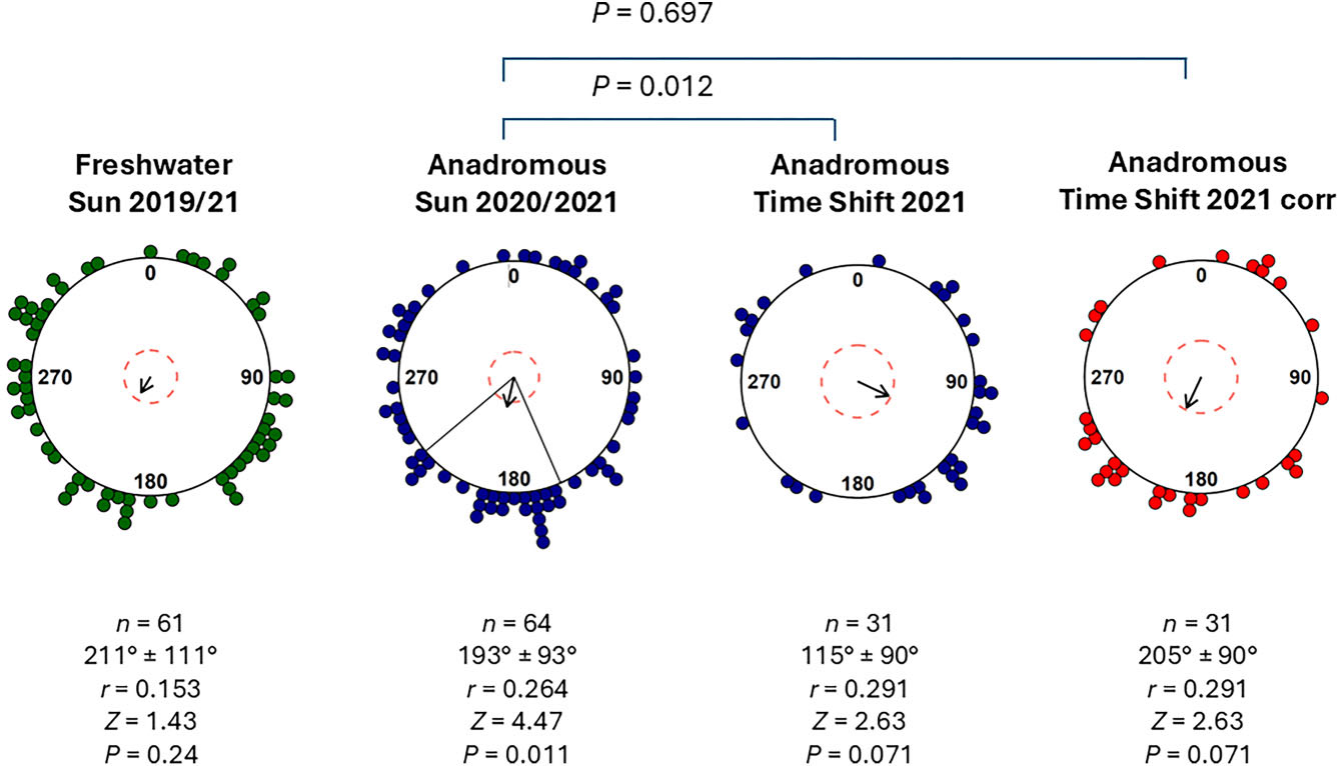
Sun compass orientation of sticklebacks. Orientation of freshwater resident (green) and anadromous sticklebacks of Sun and TS tests (both blue) are shown. We corrected for the 6-h time shift by adding 90° to each individual orientation of anadromous fish to illustrate that the TS fish corr (red) aimed for the same direction as the Sunfish. Each dot in a circular plot indicates the mean direction of one individual fish tested at least 3–6 times in the given condition. The group's mean orientation direction is indicated by the black arrow. Dashed red circles indicate the significance threshold of the Rayleigh Test (*P* < 0.05 = inner red dashed circle). Left and right of the vector arrow, solid black lines indicate the 95% confidence interval.

Freshwater resident sticklebacks caught in 2019 (*n* = 20) and 2021 (*n* = 41) did not show a significant mean directional orientation (Fig. 3). In contrast, anadromous sticklebacks sampled in 2020 (*n* = 42) and 2021 (*n* = 22) showed a statistically significant group orientation toward South, with a mean vector of 193° ± 93°SD (standard deviation); see Fig. 3. To determine whether anadromous sticklebacks primarily rely on the sun for orientation, we performed in 2021 a time-shift experiment (TS, *n* = 31). Although marginally non-significant, the TS fish orientated themselves preferentially to the South–East (mean vector = 115° ± 90°SD; see Fig. 3), which resulted in a statistically significant difference between the two datasets, Sun and TS, of 78° (Mardia–Watson–Wheeler test; *W* = 8.85, *P* = 0.012). If the fish used a time-compensated sun compass, we would expect a difference of 90° between the orientation direction of Sun and TS experiment. When we corrected the orientation by adding 90° to each individual direction of TS fish, the mean direction summed to 205°, which was consistent with the mean Sun orientation (*W* = 0.772, *P* = 0.697). Therefore, we conclude that sticklebacks can use a time-compensated sun compass.

### Impact of cloud cover

**Fig. 4.**
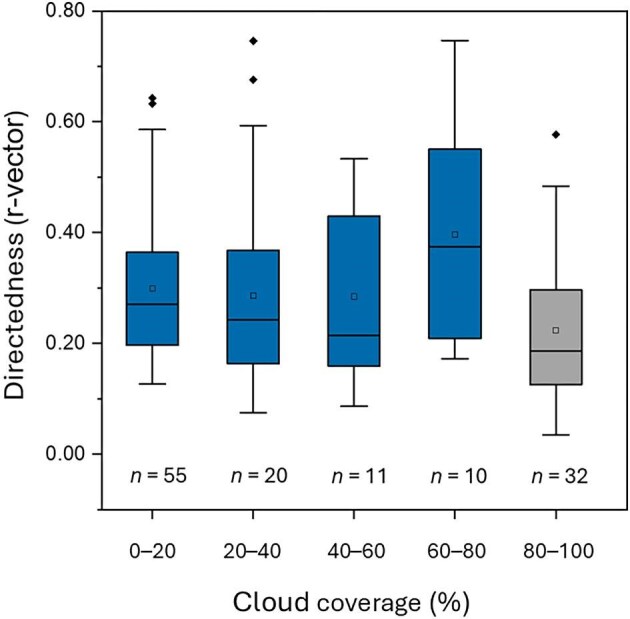
Impact of cloud coverage on orientation directedness of sticklebacks indicated by the r-vector.

If it is very cloudy, it is to be expected that orientation according to the position of the sun is not possible or at least worsens. To determine whether orientation accuracy was affected by cloud cover, we calculated the r-vector (indicating directionality) per fish and cloud cover category. As Fig. 4 and Table 1 show, orientation consistency decreased when the view of the sky was impaired by cloud cover of more than 80%.

**Table 1.** Impact of cloud coverage on the directedness of animals

Effect	Group	Term	Estimate	std.error	Statistic	*P*-value
Fixed		(Intercept)	−1.44	0.16	−8.90	**0.00**
Fixed		cloud20–40%	−1.16	0.75	−1.54	0.12
Fixed		cloud40–60%	−0.26	0.79	−0.33	0.74
Fixed		cloud60–80%	−0.06	0.80	−0.08	0.94
Fixed		cloud80–100%	−2.25	0.73	−3.06	**0.00**
random	animal	sd__(Intercept)	0.00			
“quality” of model parameters						
AIC	BIC	logLik	deviance	df.resid		
306.9	330.6	−147.5	294.9	375.0		

### Different colors (gray vs. blue) of boxplots indicate statistically significant differences; see for details Table 1

A Generalized linear mixed model fit by maximum likelihood was used. Since some animals were tested repeatedly and sometimes in more than one cloud cover category, “animal” was set as a random factor and “cloud coverage” as the fixed factor. Cloud coverage had a significant effect on directedness (r-vector) (*Z* = 11.36, *df* = 4. *P* = 0.022); a significant difference could be observed between 80–100% to the other conditions.

## Discussion

Freshwater sticklebacks showed no significant orientation direction toward a common target compared to anadromous sticklebacks caught during their spawning migration at a fish pass in the river Ems. Over short distances, visual landmarks, or the direction of the water current (Braithwaite and Girvan 2003; Odling-Smee et al. 2008) have been identified as navigation strategies for freshwater sticklebacks to find back to individual habitats. In our experiments, 70% of all individually tested freshwater fish showed a significant orientation, but not as a group in the same direction, this means a high intra-individual variation was observed, which makes the existence of a sun compass appear questionable. In a former study, Ward et al. (2013) captured and tagged freshwater sticklebacks during their breeding season and transported them between 80 and 160 m into small trenches. They found their way back to the capture site during these displacement experiments. From this, we conclude that freshwater fish in contrast to anadromous sticklebacks, may not have a common goal that requires a sun compass, but show a goal-directed behavior to individual locations over short distances; a sun compass alone is unlikely to explain short-distance pattern.

It is not known from which distances anadromous sticklebacks migrate to the rivers. A time-compensated sun compass orientation could be used over long distances to find a river mouth. The coastal areas of the Wadden Sea pose some challenges for fish navigation as they are regions exposed to a strong tidal regime, strong currents, and strong energy circulation, which is likely to reduce the reliability of flow and olfactory signals for orientation. Consequently, anadromous sticklebacks may need a different or additional orientation strategy compared to freshwater sticklebacks to be guided to the river mouth. Under the Sun condition, the anadromous sticklebacks showed a clear orientation toward the South (Fig. 3B), which would lead them past the East Frisian Islands to the rivers in the South on the North German mainland. Notably, the direction of orientation relative to the sun would have to differ among populations of anadromous stickleback entering different rivers in their holarctic distribution area. The fish that were TS for 6 h, which should correspond to a 90° shift (15° per h, 6 h), showed a turn in their orientation response. The directional difference between the Sun and TS test conditions was statistically significant. Consequently, they may use a time-compensated sun compass when migrating from the sea to the rivers to spawn. Sun compass orientation is a widely exploited mechanism used by both inexperienced juvenile and experienced adult fish in temperate and tropical habitats. Orientation according to the sun compass has already been demonstrated in several fish species, for juvenile herring (*C. harengus*) (Spiecker et al. 2022), juvenile sprat (*S. sprattus*) (Laurien et al. 2024b), and dispersing juvenile coral reef fish such as *O. doederleini* (Mouritsen et al. 2013). A different experimental approach such as a DISC (drifting in-situ chamber), was used to confirm the orientation of herring larvae according to the sun compass (Cresci et al. 2020). As further evidence that the anadromous sticklebacks use the view of the sun for orientation, we consider the deterioration in orientation accuracy with increasing cloud cover. When the sky was more than 80% covered with clouds, the sticklebacks orientated themselves significantly worse. We obtained similar results when we tested the influence of cloud cover on herring (*C. harengus*) (Spiecker et al. 2022) and sprats (*S. sprattus*) (Laurien et al. 2024b). The orientation consistency could be different if the fish had been tested in groups, as they migrate in groups. Modeling approaches support this assumption that groups show a higher accuracy in orientation than individually tested animals of the same species (Berdahl et al. 2016; Berdahl et al. 2018).

TS fish showed a turn of 78° but not 90°; this could also indicate that a mismatch in the sensory signals (cue conflict) might interfere with the directedness of the fish and cause this difference. For example, if sticklebacks had a magnetic compass, its information would not match the position of the sun in the TS condition. This discrepancy between the celestial information (indicating an easterly direction) and the magnetic compass information (still indicating a southerly direction) could explain the incomplete turn of the TS fish to the time-shifted position of the sun. A magnetic compass as it has been shown in herring (Laurien et al. 2024a), cardinal fish (*O. doederleini*) (Bottesch et al. 2016), and eel (Cresci et al. 2017) could be involved. Using the earth magnetic field for orientation would even allow nocturnal orientation (Bottesch et al. 2016); however, in sticklebacks this has not been evaluated yet. If sticklebacks return to their natal rivers is also not known so far and would require a multi river sampling over several years assuming that natal homing would result in a higher genetic similarity within a river population in consecutive years compared to populations from different rivers.

## Supplementary Material

obaf022_Supplemental_File

## Data Availability

Raw data are included in Supplementary data

## References

[bib1] Aguirre WE, Reid K, Rivera J, Heins DC, Veeramah KR, Bell MA. 2022. Freshwater colonization, adaptation, and genomic divergence in threespine stickleback. Integr Comp Biol 62:388–405. 10.1093/icb/icac071 35660873 PMC9405723

[bib2] Archambeault SL, Bärtschi LR, Merminod AD, Peichel CL. 2020. Adaptation via pleiotropy and linkage: association mapping reveals a complex genetic architecture within the stickleback *Eda* locus. Evol Lett 4:282–301. 10.1002/evl3.175 32774879 PMC7403726

[bib3] Atema J, Kingsford MJ, Gerlach G. 2002. Larval reef fish could use odour for detection, retention and orientation to reefs. Mar Ecol Prog Ser 241:151–60. 10.3354/meps241151

[bib4] Barrett RDH . 2010. Adaptive evolution of lateral plates in three-spined stickleback *Gasterosteus aculeatus*: a case study in functional analysis of natural variation. J Fish Biol 77:311–28. 10.1111/j.1095-8649.2010.02640.x 20646158

[bib5] Barrett RDH, Rogers SM, Schluter D. 2008. Natural selection on a major armor gene in threespine stickleback. Science 322:255–7. 10.1126/science.1159978 18755942

[bib6] Berdahl A, Westley PAH, Levin SA, Couzin ID, Quinn TP. 2016. A collective navigation hypothesis for homeward migration in anadromous salmonids. Fish Fish 17:525–42. 10.1111/faf.12084

[bib7] Berdahl AM, Kao AB, Flack A, Westley PAH, Codling EA, Couzin ID, Dell AI, Biro D. 2018. Collective animal navigation and migratory culture: from theoretical models to empirical evidence. Phil Trans Roy Soc B 373:20170009. 10.1098/rstb.2017.0009 29581394 PMC5882979

[bib8] Bottesch M, Gerlach G, Halbach M, Bally A, Kingsford MJ, Mouritsen H. 2016. A magnetic compass that might help coral reef fish larvae return to their natal reef. Curr Biol 26:R1266–7. 10.1016/j.cub.2016.10.051 27997833

[bib8a] Braithwaite VA, Girvan JR. 2003. Use of water flow direction to provide spatial information in a small-scale orientation task. J Fish Biol 63:74–83. 10.1111/j.1095-8649.2003.00218.x

[bib9] Colosimo PF, Hosemann KE, Balabhadra S, Villarreal G, Dickson M, Grimwood J, Schmutz J, Myers RM, Schluter D, Kingsley DM. 2005. Widespread parallel evolution in sticklebacks by repeated fixation of ectodysplasin alleles. Science 307:1928–33. 10.1126/science.1107239 15790847

[bib10] Colosimo PF, Peichel CL, Nereng K, Blackman BK, Shapiro MD, Schluter D, Kingsley DM. 2004. The genetic architecture of parallel armor plate reduction in threespine sticklebacks. PloS Biol 2:e109. 10.1371/journal.pbio.0020109 15069472 PMC385219

[bib11] Cresci A, Allan BJM, Shema SD, Skiftesvik AB, Browman HI. 2020. Orientation behavior and swimming speed of Atlantic herring larvae (*Clupea harengus*) in situ and in laboratory exposures to rotated artificial magnetic fields. J Exp Mar Biol Ecol 526:151358. 10.1016/j.jembe.2020.151358

[bib12] Cresci A, Paris CB, Durif CMF, Shema S, Bjelland RM, Skiftesvik AB, Browman HI. 2017. Glass eels (*Anguilla anguilla*) have a magnetic compass linked to the tidal cycle. Sci Adv 3:e1602007. 10.1126/sciadv.1602007. 28630895 PMC5466372

[bib13] Fisher NI . 1993. Statistical analysis of circular data. Cambridge: Cambridge University Press

[bib14] Formicki K, Korzelecka-Orkisz A, Tanski A. 2019. Magnetoreception in fish. J Fish Biol 95:73–91. 10.1111/jfb.13998 31054161

[bib15] Gerlach G, Atema J, Kingsford MJ, Black KP, Miller-Sims V. 2007. Smelling home can prevent dispersal of reef fish larvae. Proc Natl Acad Sci U S A 104:858–63. 10.1073/pnas.0606777104 17213323 PMC1783404

[bib16] Hansen MJ, Ward AJW, Furtbauer I, King AJ. 2016. Environmental quality determines finder-joiner dynamics in socially foraging three-spined sticklebacks (*Gasterosteus aculeatus*). Behav Ecol Sociobiol 70:889–99. 10.1007/s00265-016-2111-5

[bib17] Hasler AD, Wisby WJ. 1951. Discrimination of stream odors by fishes and relation to parent stream behavior. Am Nat 85:223–38.

[bib18] Kovach WL 2011. Oriana—circular statistics for windows. Pentraeth, Wales, U.K.: Kovach Computing Services.

[bib19] Laurien M, Mende L, Luhrmann L, Frederiksen A, Aldag M, Spiecker L, Clemmesen C, Solov'yov IA, Gerlach G. 2024a. Magnetic orientation in juvenile Atlantic herring (*Clupea harengus*) could involve cryptochrome 4 as a potential magnetoreceptor. J R Soc Interface 21:20240035. 10.1098/rsif.2024.0035 38835248 PMC11285480

[bib20] Laurien M, Spiecker L, Luhrmann L, Mende L, Dammann W, Clemmesen C, Gerlach G. 2024b. Time-compensated sun compass in juvenile sprat (*Sprattus sprattus*) reveals the onset of migratory readiness. J Exp Biol 227:jeb246188. 10.1242/jeb.246188 38291981

[bib21] McKinnon JS, Rundle HD. 2002. Speciation in nature: the threespine stickleback model systems. Trends Ecol Evol 17:480–8.

[bib22] Mouritsen H, Atema J, Kingsford MJ, Gerlach G. 2013. Sun compass orientation helps coral reef fish larvae return to their natal reef. PLoS ONE 8:e66039. 10.1371/journal.pone.0066039 23840396 PMC3694079

[bib23] Myhre F, Klepaker T. 2009. Body armour and lateral-plate reduction in freshwater three–spined stickleback *Gasterosteus aculeatus*: adaptations to a different buoyancy regime? J Fish Biol 75:2062–74. 10.1111/j.1095-8649.2009.02404.x 20738672

[bib24a] Odling-Smee LC, Boughman JW, Braithwaite VA et al. 2008. Sympatric species of threespine stickleback differ in their performance in a spatial learning task. Behav Ecol Sociobiol 62:1935–45.

[bib25] Radford CA, Stanley JA, Simpson SD, Jeffs AG. 2011. Juvenile coral reef fish use sound to locate habitats. Coral Reefs 30:295–305. 10.1007/s00338-010-0710-6

[bib27] Rundle HD, Nagel L, Wenrick Boughman J, Schluter D. 2000. Natural selection and parallel speciation in sympatric sticklebacks. Science 287:306–8. 10634785 10.1126/science.287.5451.306

[bib28] Schluter D, Clifford Elizabeth A, Nemethy M, McKinnon Jeffrey S. 2004. Parallel evolution and inheritance of quantitative traits. Am Nat 163:809–22. 10.1086/383621 15266380

[bib30] Spiecker L, Laurien M, Dammann W, Franke A, Clemmesen C, Gerlach G. 2022. Juvenile Atlantic herring (*Clupea harengus*) use a time-compensated sun compass for orientation. J Exp Biol 225: jeb.244607. 10.1242/jeb.244607 35996951

[bib31] Walker JA . 2008. Ecological morphology of lacustrine threespine stickleback *Gasterosteus aculeatus* L. (Gasterosteidae) body shape. Biol J Linn Soc Lond 61:3–50. 10.1111/j.1095-8312.1997.tb01777.x

[bib32] Ward AJW, James R, Wilson ADM, Webster MM. 2013. Site fidelity and localised homing behaviour in three-spined sticklebacks (*Gasterosteus aculeatus*). Behaviour 150:1689–708. 10.1163/1568539X-00003115

[bib33] Wickham H, Chang W, Henry L, Pedersen T, Takahashi K, Wilke C, Woo K, Yutani J, Dunnington D, Brand TVD, Posit Software, & PBC. 2024. ggplot2: create elegant data visualisations using the grammar of graphics. https://cran.r-project.org/web/packages/ggplot2/index.html.

[bib34] Wootton RJ . 1976. The biology of the sticklebacks. London: Academic Press. 387.

