# Supplementary material for: Comparative Study of Sun Compass Orientation in Migrating Anadromous versus Resident Freshwater Threespine Sticklebacks (Gasterosteus aculeatus)
Source: Integr Org Biol. 2025 May 28;7(1):obaf022. doi: 10.1093/iob/obaf022 (PMC12152475; doi:10.1093/iob/obaf022)
Supplement: obaf022_Supplemental_File [file obaf022_supplemental_file.docx]

**Supplementary data:**

Supplemental Tables:

**Table S1:** Raw data of orientation tests Freshwater sticklebacks Löninger Mühlenbach 2019 and 2021 Sun - including all experimental runs

| FreshwaterF |  |  |  |  |  |  |  |  |
| --- | --- | --- | --- | --- | --- | --- | --- | --- |
| Year | Fish # | Number of Observations | Mean Vector (µ) | Length of Mean Vector (r) | Circular Variance | Circular Standard Deviation | Rayleigh Test (Z) | Rayleigh Test (p) |
| 2019 | 1 | 3 | 296 | 0.499 | 0.501 | 68 | 0.746 | 0.516 |
| 2019 | 2 | 3 | 201 | 0.973 | 0.027 | 14 | 2.838 | 0.043 |
| 2019 | 3 | 3 | 18 | 0.787 | 0.213 | 40 | 1.860 | 0.161 |
| 2019 | 4 | 3 | 314 | 0.726 | 0.274 | 46 | 1.580 | 0.221 |
| 2019 | 5 | 3 | 100 | 0.364 | 0.636 | 81 | 0.398 | 0.709 |
| 2019 | 6 | 3 | 171 | 0.344 | 0.656 | 84 | 0.356 | 0.736 |
| 2019 | 7 | 3 | 301 | 0.943 | 0.057 | 20 | 2.668 | 0.056 |
| 2019 | 8 | 3 | 295 | 0.395 | 0.605 | 78 | 0.467 | 0.667 |
| 2019 | 9 | 3 | 217 | 0.829 | 0.171 | 35 | 2.060 | 0.126 |
| 2019 | 10 | 3 | 132 | 0.918 | 0.082 | 24 | 2.531 | 0.068 |
| 2019 | 11 | 3 | 336 | 0.219 | 0.781 | 100 | 0.144 | 0.885 |
| 2019 | 12 | 3 | 92 | 0.305 | 0.695 | 88 | 0.279 | 0.787 |
| 2019 | 13 | 3 | 24 | 0.31 | 0.69 | 88 | 0.288 | 0.781 |
| 2019 | 14 | 3 | 268 | 0.449 | 0.551 | 73 | 0.604 | 0.589 |
| 2019 | 15 | 3 | 263 | 0.546 | 0.454 | 63 | 0.895 | 0.448 |
| 2019 | 16 | 3 | 244 | 0.306 | 0.694 | 88 | 0.281 | 0.786 |
| 2019 | 17 | 3 | 215 | 0.62 | 0.38 | 56 | 1.154 | 0.347 |
| 2019 | 18 | 3 | 100 | 0.303 | 0.697 | 88 | 0.276 | 0.789 |
| 2019 | 19 | 3 | 15 | 0.512 | 0.488 | 66 | 0.787 | 0.497 |
| 2019 | 20 | 3 | 56 | 0.41 | 0.59 | 77 | 0.504 | 0.645 |
| 2021 | 1 | 6 | 273 | 0.392 | 0.608 | 78 | 0.922 | 0.416 |
| 2021 | 2 | 6 | 273 | 0.353 | 0.647 | 83 | 0.747 | 0.494 |
| 2021 | 3 | 6 | 111 | 0.627 | 0.373 | 55 | 2.359 | 0.091 |
| 2021 | 4 | 6 | 267 | 0.399 | 0.601 | 78 | 0.955 | 0.402 |
| 2021 | 5 | 6 | 301 | 0.603 | 0.397 | 58 | 2.182 | 0.111 |
| 2021 | 6 | 6 | 212 | 0.581 | 0.419 | 60 | 2.028 | 0.132 |
| 2021 | 7 | 6 | 126 | 0.623 | 0.377 | 56 | 2.329 | 0.094 |
| 2021 | 8 | 6 | 35 | 0.552 | 0.448 | 62 | 1.828 | 0.163 |
| 2021 | 9 | 6 | 234 | 0.167 | 0.833 | 108 | 0.167 | 0.857 |
| 2021 | 10 | 6 | 253 | 0.434 | 0.566 | 74 | 1.131 | 0.337 |
| 2021 | 11 | 6 | 303 | 0.383 | 0.617 | 79 | 0.881 | 0.433 |
| 2021 | 12 | 6 | 146 | 0.789 | 0.211 | 39 | 3.737 | 0.016 |
| 2021 | 13 | 6 | 122 | 0.744 | 0.256 | 44 | 3.322 | 0.028 |
| 2021 | 14 | 6 | 256 | 0.507 | 0.493 | 67 | 1.543 | 0.221 |
| 2021 | 15 | 6 | 2 | 0.034 | 0.966 | 149 | 0.007 | 0.994 |
| 2021 | 16 | 6 | 315 | 0.94 | 0.06 | 20 | 5.301 | 0.001 |
| 2021 | 17 | 6 | 200 | 0.505 | 0.495 | 67 | 1.533 | 0.223 |
| 2021 | 18 | 6 | 145 | 0.8 | 0.2 | 38 | 3.839 | 0.014 |
| 2021 | 20 | 6 | 329 | 0.925 | 0.075 | 23 | 5.130 | 0.002 |
| 2021 | 21 | 6 | 136 | 0.868 | 0.132 | 30 | 4.520 | 0.005 |
| 2021 | 22 | 6 | 190 | 0.633 | 0.367 | 55 | 2.408 | 0.086 |
| 2021 | 23 | 6 | 121 | 0.554 | 0.446 | 62 | 1.841 | 0.161 |
| 2021 | 24 | 6 | 289 | 0.648 | 0.352 | 53 | 2.516 | 0.076 |
| 2021 | 25 | 6 | 190 | 0.537 | 0.463 | 64 | 1.731 | 0.181 |
| 2021 | 26 | 5 | 295 | 0.68 | 0.32 | 50 | 2.312 | 0.095 |
| 2021 | 27 | 6 | 191 | 0.348 | 0.652 | 83 | 0.726 | 0.504 |
| 2021 | 28 | 6 | 213 | 0.303 | 0.697 | 89 | 0.550 | 0.597 |
| 2021 | 29 | 6 | 126 | 0.704 | 0.296 | 48 | 2.971 | 0.044 |
| 2021 | 30 | 6 | 180 | 0.45 | 0.55 | 72 | 1.212 | 0.311 |
| 2021 | 31 | 4 | 229 | 0.243 | 0.757 | 96 | 0.237 | 0.81 |
| 2021 | 32 | 6 | 302 | 0.846 | 0.154 | 33 | 4.294 | 0.007 |
| 2021 | 33 | 6 | 114 | 0.898 | 0.102 | 27 | 4.844 | 0.003 |
| 2021 | 34 | 6 | 138 | 0.35 | 0.65 | 83 | 0.733 | 0.5 |
| 2021 | 35 | 6 | 113 | 0.269 | 0.731 | 93 | 0.436 | 0.666 |
| 2021 | 36 | 6 | 146 | 0.627 | 0.373 | 55 | 2.361 | 0.091 |
| 2021 | 37 | 6 | 193 | 0.381 | 0.619 | 80 | 0.872 | 0.437 |
| 2021 | 38 | 6 | 34 | 0.123 | 0.877 | 117 | 0.091 | 0.919 |
| 2021 | 39 | 6 | 90 | 0.458 | 0.542 | 72 | 1.256 | 0.297 |
| 2021 | 40 | 6 | 259 | 0.323 | 0.677 | 86 | 0.626 | 0.555 |
| 2021 | 48 | 6 | 56 | 0.34 | 0.66 | 84 | 0.695 | 0.519 |
| 2021 | 50 | 6 | 62 | 0.349 | 0.651 | 83 | 0.731 | 0.501 |

| Anadromous sticklebacks Sun |  |  |  |  |  |  |  |  |
| --- | --- | --- | --- | --- | --- | --- | --- | --- |
| Year | Fish# | Number of Observations | Mean Vector (µ) | Length of Mean Vector (r) | Circular Variance | Circular Standard Deviation | Rayleigh Test (Z) | Rayleigh Test (p) |
| 2020 | 1 | 6 | 223 | 0.196 | 0.804 | 103 | 0.23 | 0.808 |
| 2020 | 2 | 6 | 278 | 0.528 | 0.472 | 65 | 1.672 | 0.193 |
| 2020 | 3 | 4 | 191 | 0.582 | 0.418 | 60 | 1.353 | 0.276 |
| 2020 | 4 | 5 | 241 | 0.489 | 0.511 | 69 | 1.196 | 0.319 |
| 2020 | 5 | 6 | 6 | 0.403 | 0.597 | 77 | 0.973 | 0.395 |
| 2020 | 6 | 6 | 196 | 0.069 | 0.931 | 132 | 0.029 | 0.974 |
| 2020 | 7 | 4 | 169 | 0.884 | 0.116 | 28 | 3.125 | 0.032 |
| 2020 | 8 | 6 | 232 | 0.824 | 0.176 | 36 | 4.07 | 0.01 |
| 2020 | 9 | 6 | 52 | 0.185 | 0.815 | 105 | 0.205 | 0.827 |
| 2020 | 10 | 6 | 49 | 0.272 | 0.728 | 92 | 0.446 | 0.660 |
| 2020 | 11 | 6 | 160 | 0.328 | 0.672 | 86 | 0.647 | 0.544 |
| 2020 | 12 | 6 | 187 | 0.704 | 0.296 | 48 | 2.976 | 0.044 |
| 2020 | 13 | 4 | 181 | 0.234 | 0.766 | 98 | 0.220 | 0.822 |
| 2020 | 14 | 6 | 169 | 0.253 | 0.747 | 95 | 0.383 | 0.7 |
| 2020 | 15 | 6 | 136 | 0.531 | 0.469 | 64 | 1.693 | 0.189 |
| 2020 | 16 | 6 | 8 | 0.713 | 0.287 | 47 | 3.054 | 0.04 |
| 2020 | 17 | 6 | 337 | 0.426 | 0.574 | 75 | 1.087 | 0.353 |
| 2020 | 18 | 6 | 168 | 0.285 | 0.715 | 91 | 0.487 | 0.634 |
| 2020 | 19 | 6 | 197 | 0.844 | 0.156 | 33 | 4.279 | 0.007 |
| 2020 | 20 | 6 | 32 | 0.585 | 0.415 | 59 | 2.051 | 0.128 |
| 2020 | 21 | 6 | 112 | 0.762 | 0.238 | 42 | 3.481 | 0.023 |
| 2020 | 22 | 6 | 246 | 0.282 | 0.718 | 91 | 0.476 | 0.641 |
| 2020 | 23 | 6 | 169 | 0.952 | 0.048 | 18 | 5.433 | 7.29E-04 |
| 2020 | 24 | 6 | 100 | 0.478 | 0.522 | 70 | 1.373 | 0.264 |
| 2020 | 25 | 6 | 160 | 0.447 | 0.553 | 73 | 1.198 | 0.315 |
| 2020 | 26 | 6 | 224 | 0.293 | 0.707 | 90 | 0.515 | 0.617 |
| 2020 | 27 | 6 | 213 | 0.525 | 0.475 | 65 | 1.651 | 0.197 |
| 2020 | 28 | 6 | 54 | 0.714 | 0.286 | 47 | 3.055 | 0.04 |
| 2020 | 29 | 6 | 251 | 0.508 | 0.492 | 67 | 1.548 | 0.22 |
| 2020 | 30 | 6 | 175 | 0.283 | 0.717 | 91 | 0.479 | 0.639 |
| 2020 | 31 | 4 | 134 | 0.922 | 0.078 | 23 | 3.400 | 0.021 |
| 2020 | 32 | 6 | 166 | 0.504 | 0.496 | 67 | 1.523 | 0.226 |
| 2020 | 33 | 6 | 163 | 0.666 | 0.334 | 52 | 2.661 | 0.064 |
| 2020 | 34 | 6 | 225 | 0.757 | 0.243 | 43 | 3.436 | 0.024 |
| 2020 | 35 | 6 | 168 | 0.463 | 0.537 | 71 | 1.285 | 0.289 |
| 2020 | 36 | 6 | 184 | 0.591 | 0.409 | 59 | 2.096 | 0.122 |
| 2020 | 37 | 6 | 21 | 0.404 | 0.596 | 77 | 0.981 | 0.392 |
| 2020 | 38 | 6 | 107 | 0.281 | 0.719 | 91 | 0.473 | 0.642 |
| 2020 | 39 | 6 | 88 | 0.59 | 0.41 | 59 | 2.089 | 0.123 |
| 2020 | 40 | 6 | 302 | 0.448 | 0.552 | 73 | 1.204 | 0.314 |
| 2020 | 41 | 6 | 253 | 0.187 | 0.813 | 105 | 0.210 | 0.823 |
| 2020 | 42 | 6 | 39 | 0.518 | 0.482 | 66 | 1.611 | 0.206 |
| 2021 | 43 | 6 | 134 | 0.425 | 0.575 | 75 | 1.082 | 0.354 |
| 2021 | 44 | 6 | 23 | 0.446 | 0.554 | 73 | 1.194 | 0.317 |
| 2021 | 45 | 6 | 124 | 0.705 | 0.295 | 48 | 2.982 | 0.044 |
| 2021 | 46 | 6 | 141 | 0.933 | 0.067 | 21 | 5.225 | 0.001 |
| 2021 | 47 | 6 | 267 | 0.031 | 0.969 | 151 | 0.006 | 0.995 |
| 2021 | 49 | 6 | 302 | 0.915 | 0.085 | 24 | 5.024 | 0.002 |
| 2021 | 51 | 6 | 355 | 0.485 | 0.515 | 69 | 1.412 | 0.253 |
| 2021 | 52 | 6 | 78 | 0.48 | 0.52 | 69 | 1.38 | 0.262 |
| 2021 | 53 | 6 | 281 | 0.796 | 0.204 | 39 | 3.805 | 0.015 |
| 2021 | 54 | 6 | 232 | 0.056 | 0.944 | 138 | 0.018 | 0.983 |
| 2021 | 55 | 6 | 28 | 0.611 | 0.389 | 57 | 2.238 | 0.104 |
| 2021 | 56 | 6 | 150 | 0.32 | 0.68 | 87 | 0.613 | 0.562 |
| 2021 | 57 | 6 | 196 | 0.967 | 0.033 | 15 | 5.616 | 3.92E-04 |
| 2021 | 58 | 6 | 315 | 0.856 | 0.144 | 32 | 4.399 | 0.006 |
| 2021 | 59 | 6 | 291 | 0.938 | 0.062 | 21 | 5.274 | 0.001 |
| 2021 | 60 | 6 | 295 | 0.059 | 0.941 | 137 | 0.021 | 0.981 |
| 2021 | 61 | 6 | 290 | 0.577 | 0.423 | 60 | 2.000 | 0.136 |
| 2021 | 62 | 6 | 307 | 0.286 | 0.714 | 91 | 0.492 | 0.631 |
| 2021 | 63 | 6 | 205 | 0.331 | 0.669 | 85 | 0.659 | 0.538 |
| 2021 | 64 | 6 | 174 | 0.67 | 0.33 | 51 | 2.697 | 0.061 |
| 2021 | 65 | 6 | 253 | 0.675 | 0.325 | 51 | 2.736 | 0.059 |
| 2021 | 66 | 6 | 192 | 0.505 | 0.495 | 67 | 1.532 | 0.224 |

| Anadromous sticklebacks Timeshift TS |  |  |  |  |  |  |  |  |  |
| --- | --- | --- | --- | --- | --- | --- | --- | --- | --- |
| Year | Fish# | Number of Observations | Mean Vector (µ) | corrected 90° | Length of Mean Vector (r) | Circular Variance | Circular Standard Deviation | Rayleigh Test (Z) | Rayleigh Test (p) |
| 2021 | 1 | 6 | 135 | 225 | 0.63 | 0.37 | 55.101 | 2.379 | 0.089 |
| 2021 | 2 | 6 | 137 | 227 | 0.758 | 0.242 | 42.623 | 3.450 | 0.024 |
| 2021 | 3 | 6 | 250 | 340 | 0.665 | 0.335 | 51.769 | 2.652 | 0.065 |
| 2021 | 5 | 6 | 9 | 99 | 0.319 | 0.681 | 86.554 | 0.612 | 0.562 |
| 2021 | 6 | 6 | 149 | 239 | 0.662 | 0.338 | 52.018 | 2.631 | 0.066 |
| 2021 | 7 | 6 | 71 | 161 | 0.171 | 0.829 | 107.649 | 0.176 | 0.85 |
| 2021 | 8 | 6 | 105 | 195 | 0.415 | 0.585 | 76.01 | 1.032 | 0.372 |
| 2021 | 11 | 6 | 132 | 222 | 0.552 | 0.448 | 62.434 | 1.83 | 0.163 |
| 2021 | 12 | 6 | 156 | 246 | 0.725 | 0.275 | 45.947 | 3.154 | 0.035 |
| 2021 | 13 | 6 | 312 | 42 | 0.291 | 0.709 | 89.993 | 0.509 | 0.621 |
| 2021 | 14 | 6 | 61 | 151 | 0.218 | 0.782 | 100.005 | 0.285 | 0.767 |
| 2021 | 15 | 6 | 95 | 185 | 0.389 | 0.611 | 78.714 | 0.909 | 0.421 |
| 2021 | 16 | 6 | 135 | 225 | 0.912 | 0.088 | 24.596 | 4.990 | 0.002 |
| 2021 | 90 | 6 | 213 | 303 | 0.369 | 0.631 | 80.888 | 0.818 | 0.461 |
| 2021 | 91 | 6 | 40 | 130 | 0.105 | 0.895 | 121.707 | 0.066 | 0.941 |
| 2021 | 92 | 6 | 202 | 292 | 0.414 | 0.586 | 76.072 | 1.029 | 0.374 |
| 2021 | 93 | 6 | 159 | 249 | 0.571 | 0.429 | 60.691 | 1.954 | 0.143 |
| 2021 | 94 | 6 | 47 | 137 | 0.481 | 0.519 | 69.272 | 1.391 | 0.259 |
| 2021 | 97 | 6 | 302 | 32 | 0.384 | 0.616 | 79.288 | 0.884 | 0.431 |
| 2021 | 98 | 6 | 109 | 199 | 0.831 | 0.169 | 34.813 | 4.148 | 0.009 |
| 2021 | 99 | 6 | 93 | 183 | 0.614 | 0.386 | 56.636 | 2.258 | 0.102 |
| 2021 | 101 | 6 | 111 | 201 | 0.867 | 0.133 | 30.576 | 4.513 | 0.005 |
| 2021 | 102 | 6 | 334 | 64 | 0.61 | 0.39 | 56.929 | 2.236 | 0.104 |
| 2021 | 104 | 6 | 210 | 300 | 0.404 | 0.596 | 77.186 | 0.977 | 0.393 |
| 2021 | 105 | 6 | 88 | 178 | 0.326 | 0.674 | 85.792 | 0.637 | 0.549 |
| 2021 | 106 | 6 | 129 | 219 | 0.67 | 0.33 | 51.254 | 2.695 | 0.061 |
| 2021 | 107 | 6 | 297 | 27 | 0.862 | 0.138 | 31.207 | 4.460 | 0.006 |
| 2021 | 109 | 6 | 278 | 8 | 0.472 | 0.528 | 70.24 | 1.335 | 0.274 |
| 2021 | 110 | 6 | 44 | 134 | 0.103 | 0.897 | 122.21 | 0.063 | 0.943 |
| 2021 | 113 | 6 | 149 | 239 | 0.467 | 0.533 | 70.727 | 1.307 | 0.282 |
